# Automatic segmentation of 15 critical anatomical labels and measurements of cardiac axis and cardiothoracic ratio in fetal four chambers using nnU-NetV2

**DOI:** 10.1186/s12911-024-02527-x

**Published:** 2024-05-21

**Authors:** Bocheng Liang, Fengfeng Peng, Dandan Luo, Qing Zeng, Huaxuan Wen, Bowen Zheng, Zhiying Zou, Liting An, Huiying Wen, Xin Wen, Yimei Liao, Ying Yuan, Shengli Li

**Affiliations:** 1 Department of Ultrasound, Shenzhen Maternity&Child Healthcare Hospital, Shenzhen, 518028 China; 2 Department of Computer Science and Electronic Engineering, Hunan University, Changsha, 410082 China

**Keywords:** Ultrasound, Deep learning, Fetal, Four-chamber view

## Abstract

**Background:**

Accurate segmentation of critical anatomical structures in fetal four-chamber view images is essential for the early detection of congenital heart defects. Current prenatal screening methods rely on manual measurements, which are time-consuming and prone to inter-observer variability. This study develops an AI-based model using the state-of-the-art nnU-NetV2 architecture for automatic segmentation and measurement of key anatomical structures in fetal four-chamber view images.

**Methods:**

A dataset, consisting of 1,083 high-quality fetal four-chamber view images, was annotated with 15 critical anatomical labels and divided into training/validation (867 images) and test (216 images) sets. An AI-based model using the nnU-NetV2 architecture was trained on the annotated images and evaluated using the mean Dice coefficient (mDice) and mean intersection over union (mIoU) metrics. The model’s performance in automatically computing the cardiac axis (CAx) and cardiothoracic ratio (CTR) was compared with measurements from sonographers with varying levels of experience.

**Results:**

The AI-based model achieved a mDice coefficient of 87.11% and an mIoU of 77.68% for the segmentation of critical anatomical structures. The model’s automated CAx and CTR measurements showed strong agreement with those of experienced sonographers, with respective intraclass correlation coefficients (ICCs) of 0.83 and 0.81. Bland–Altman analysis further confirmed the high agreement between the model and experienced sonographers.

**Conclusion:**

We developed an AI-based model using the nnU-NetV2 architecture for accurate segmentation and automated measurement of critical anatomical structures in fetal four-chamber view images. Our model demonstrated high segmentation accuracy and strong agreement with experienced sonographers in computing clinically relevant parameters. This approach has the potential to improve the efficiency and reliability of prenatal cardiac screening, ultimately contributing to the early detection of congenital heart defects.

## Introduction

**Fig. 1:**
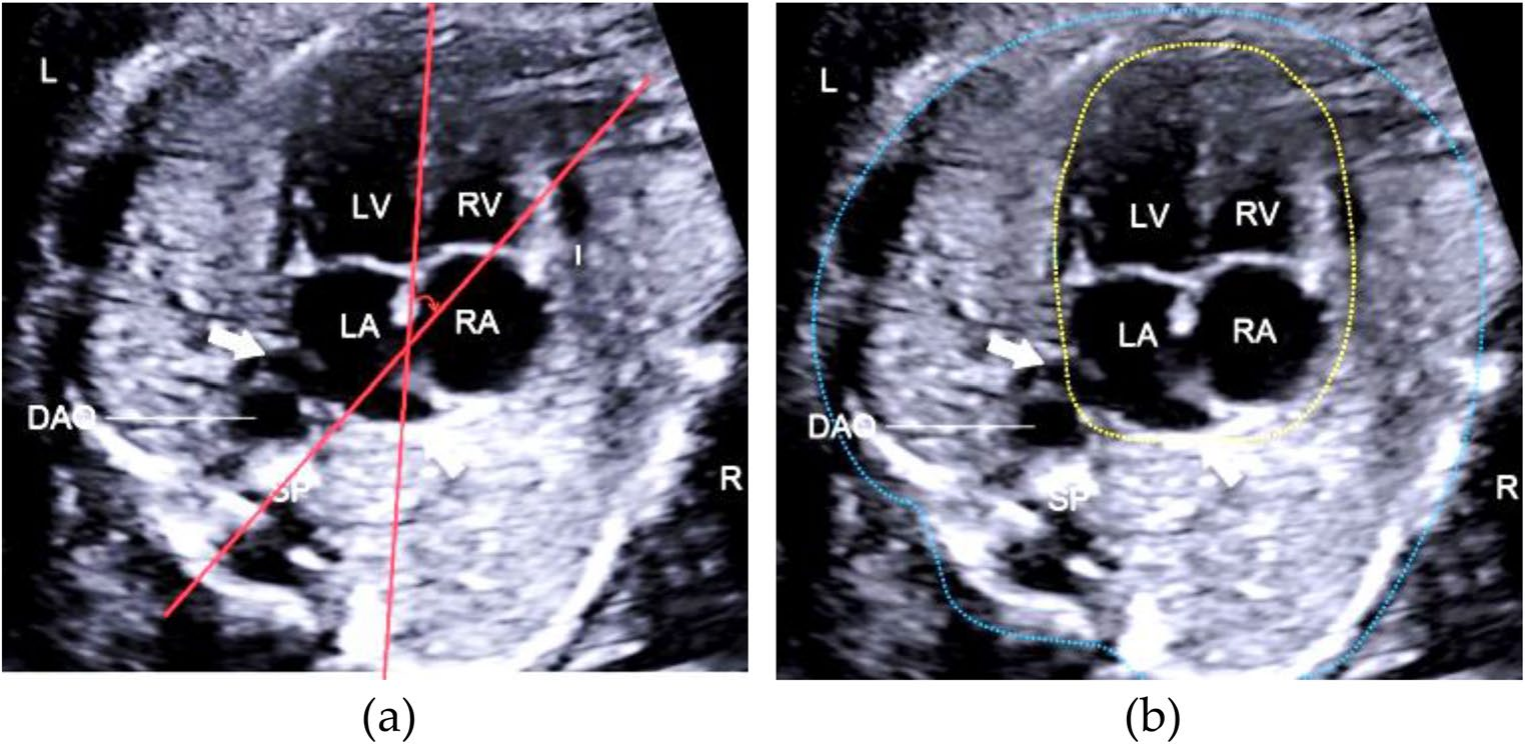
Example illustrating manual delineation measurements. (**a**) Cardiac axis measurement: the angle between red lines denotes the cardiac axis; (**b**) Cardiothoracic ratio measurement: the yellow dashed area signifies the cardiac area, the blue dashed area indicates the thoracic region, and the ratio of the heart area to the chest area is the cardiothoracic ratio. *LV: left ventricle; LA: left atrium; RA: right atrium; RV: right ventricle; DAO: descending aorta; SP: spine

Fetal echocardiography is a crucial tool in prenatal care, allowing for the assessment of fetal cardiac anatomy and function [1]. The four-chamber view is one of the most important in fetal echocardiography, providing valuable information for the detection of congenital heart defects (CHDs) [2]. Current guidelines recommend the use of the fetal cardiac axis (CAx) and cardiothoracic ratio (CTR) as key metrics for evaluating cardiac position and function [3, 4]. The CAx is determined by drawing a line from the spine to the anterior chest wall, bisecting the thorax into symmetrical right and left sections, and drawing another line along the interventricular septum. The CAx is defined as the angle at the intersection of these two lines. The CTR is quantified using electronic calipers to measure the areas of the heart and thoracic cavity, and is calculated as the ratio of these two areas (Fig. 1). An abnormal CAx may be associated with various fetal conditions, such as cardiac outflow tract anomalies, diaphragmatic hernia, pulmonary hypoplasia, gastroschisis, and omphalocele [5]. The CTR serves as a diagnostic indicator of fetal cardiovascular status in conditions like twin-to-twin transfusion syndrome and anemia, aiding prenatal sonographers in detecting abnormalities and guiding clinical decision-making [6].

However, the accuracy and reproducibility of CAx and CTR measurements heavily depend on the sonographer’s expertise and skill level, with inter-sonographer variability being a significant concern. In clinical practice, the CAx and CTR are often evaluated longitudinally across different hospitals by sonographers of varying experience, which can introduce substantial inter-observer variability, which increases the sonographer’s workload, and may lead to heightened patient anxiety and potentially misguided clinical decisions, with serious consequences [7].

Recent advancements in deep learning and medical image processing technologies have propelled artificial intelligence (AI), with significant progress in such fields as neuroscience, fetal diagnostics and therapeutics, human emotion recognition, and the classification and quality enhancement of thyroid and breast medical images [8–25]. In the context of prenatal ultrasonography, Arnaout et al. [26] employed a U-net architecture to segment the fetal four-chamber view and calculate cardiac parameters, such as the CTR, CAx, and cardiac area change ratio, based on segmentation results [26, 27].This approach demonstrated the potential for automated computation of crucial cardiac parameters. However, the aforementioned study primarily focused on model performance, which was not compared to sonographer measurements, thus highlighting the need for further validation of its clinical utility. Furthermore, most studies integrating deep learning with prenatal ultrasonography remain at the experimental stage, lacking comparisons with sonographer measurements to establish their clinical value [2, 28–30].

The present study develops an AI-based model built on nnU-NetV2, which can automatically segment the fetal four-chamber view and measure the CAx and CTR [31]. It is expected that the model will reach the senior sonographer level, to assist junior sonographers and those in underdeveloped regions with routine screening duties. This AI-based model will not only reduce the daily workload of sonographers, but will also be able to teach inexperienced sonographers how to make proper measurements.

## Methods

### Ultrasound Imaging

The fetal four-chamber view dataset was acquired using ultrasound equipment from different manufacturers (e.g., Samsung, GE, and Philips) at our hospital. The inclusion criteria for pregnant women were a gestational age between 18 and 32 weeks and a singleton pregnancy. The exclusion criteria were suspected or known fetal congenital heart disease, declined participation, and maternal BMI ≥ 25 kg/m^2^. All fetal four-chamber view images met the image quality control requirements of ISUOG [4]. All collected images were anonymized to protect patient privacy.

### Image annotation

Eligible fetal four-chamber views were screened by three sonographers with more than 5 years of clinical experience in fetal cardiac screening. Thirteen critical structures and cardiac and thoracic areas (Table 1) were accurately labeled using UltraSonic Multi-Label (version 1.0) annotation software, which was co-developed by our team. This software facilitates the classification of image categories and bounding box detection or pixel-level segmentation of critical anatomical labels.

**Table 1:** Critical anatomical labels for fetal four-chamber view

Label	
Left Atrium	Left Ventricle
Right Atrium	Right Ventricle
Interventricular Septum	Interatrial Septum
Left Ventricular Wall	Right Ventricular Wall
Left Lung	Right Lung
Descending Aorta	Spine
RIB	Heart Area
	Thorax Area

### Model training

In the present study, we employed the recently proposed nnU-NetV2 framework (version 2.0), which is an updated version of the original nnU-Net architecture [31], specifically designed for medical image segmentation. The nnU-NetV2 framework was implemented using PyTorch (version 2.1.0) and Python (version 3.9.0). The hyperparameters used by nnUNetv2 are shown in Table 2.

**Table 2:** Hyperparameter settings in experiment

Initial learning	1e-2
Weight decay	3e-5
Oversample foreground percent	0.33
Epoch	100

**Fig. 2:**
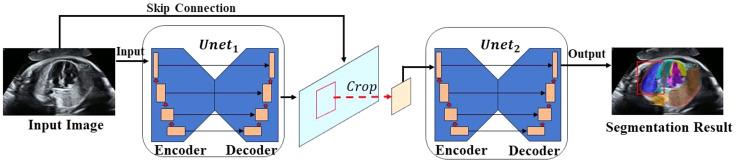
Architecture of nnU-NetV2 model

The nnU-NetV2 model has a U-shaped architecture designed to seamlessly integrate high-level semantic features with low-level detailed features:

1 $$f = Une{t_1}\left( I \right),$$

2 $${f^\prime } = Crop\left( f \right),$$

3 $$Mask\, = \,Une{t_2}\,\left( {{f^\prime }} \right),$$

where $I\in {R}^{H\times W\times C}$ is an input image, and $Crop$ is a function used to crop an image [31]. Given an input image *I*, ${Unet}_{1}$ produces features. After cropping the segmentation region from input image $I$ according to the segmentation result, the cropped image is trained on ${Unet}_{2}$ for further refinement, and the final segmentation result is obtained. Figure 2 shows the nnU-NetV2 architecture.

The training process combined Dice loss and cross-entropy loss, balancing between pixel-wise accuracy and region-based similarity, which is crucial for segmentation tasks. By leveraging the complementary strengths of these two loss functions, the aim was to achieve superior segmentation performance.

To further validate our approach, we conducted comprehensive quantitative and visual comparisons against four established semantic segmentation methods: U-Net, U-Net++, DeepLabV3+, and SAN [32–35].

The experiment was conducted on NVIDIA P100 GPUs with the PyTorch framework. Stochastic gradient descent (SGD) was utilized to optimize network performance, initiating training with a learning rate of 0.01 and a batch size of 12.

We trained nnU-NetV2 from scratch, without relying on pretrained weights, allowing tailoring to the dataset. The dataset was divided into training and validation sets at an 8:2 ratio. To ensure reliable, generalizable findings, the model was evaluated using a rigorous fivefold cross-validation approach on the training and validation sets, assessing model performance across various data subsets, for a comprehensive understanding of their predictive capabilities.

Given the challenge of training extensive neural networks with limited data, various data augmentation techniques were dynamically incorporated during training to mitigate the risk of overfitting. These included random rotations, random scaling adjustments, gamma correction for enhanced visual clarity, and mirroring. However, medical images require careful consideration of structural integrity. Hence, we avoided augmentation methods such as random elastic deformation, cutout, or other techniques potentially compromising the image structure.

### Postprocessing methods

#### CAx measurement

This study determined CAx through nnU-NetV2 segmentation masks and advanced digital image processing techniques. Specifically, the CAx was derived by fitting the interventricular septum’s long axis to the fetal thorax’s anterior-posterior axis.

A skeleton line algorithm was used to accurately determine the long axis of the interventricular septum, enabling the extraction of a set of points representing the median axis of the septum. Subsequently, a straight line was fit through these points using the least squares method, ensuring a robust and accurate representation of the long axis.

**Fig. 3:**
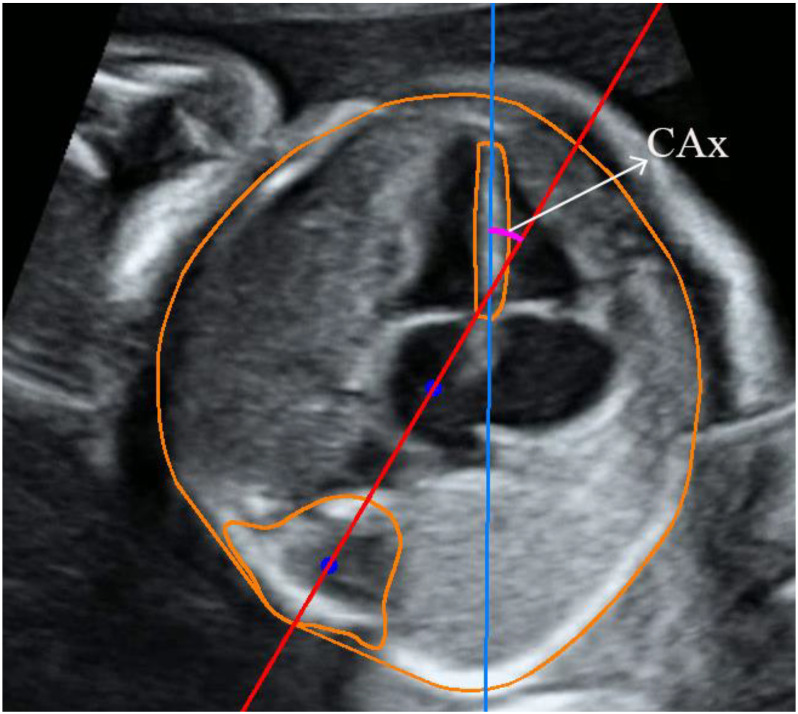
Result of cardiac axis measurement. Blue line: long axis of interventricular septum; red line: anteroposterior axis of thorax

The anterior-posterior axis of the thoracic cavity was determined using a different approach. The centers of mass lines were calculated for the thoracic and spine masks, which allowed for the precise determination of the orientation and position of the anterior-posterior axis within the thoracic cavity. The combination of these two axes established a comprehensive understanding of the CAx, which is crucial for further cardiac analysis and diagnosis. The integration of nnU-NetV2 segmentation masks and advanced digital image processing techniques has proven to be a powerful tool for enhancing the accuracy and reliability of CAx determination. The results are shown in Fig. 3.

### Measurement of cardiothoracic ratio

**Fig. 4:**
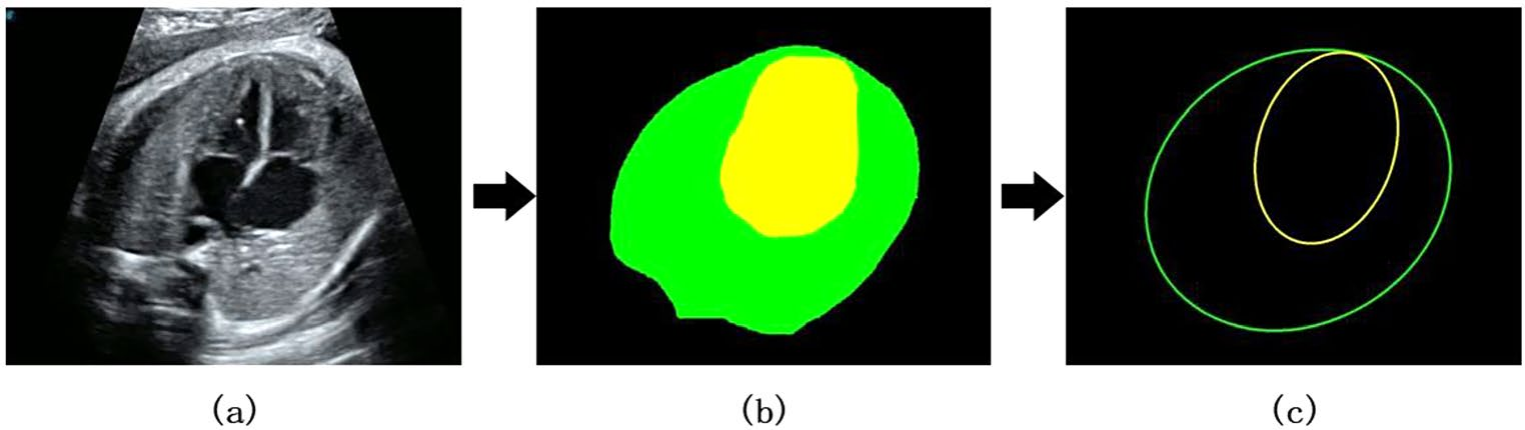
Measurement of cardiothoracic area ratio. (**a**) Original image; (**b**) Extraction of heart and chest masks; yellow: heart; green: chest; (**c**) Fitting ellipse and calculating CRT; yellow: heart; green: thoracic cavity

The CTR can be calculated from cardiac and thoracic masks, as shown in Fig. 4.

4 $${E_c} = F\left( {{m_c}} \right),$$

5 $${E_t}\, = \,F\left( {{m_t}} \right),$$

6 $$Ratio\, = \,{{A\,\left( {{E_c}} \right)} \over {A\,\left( {{E_t}} \right)}},$$

where ${m}_{c}$ and ${m}_{t}$ refer to the heart mask map and chest mask map, respectively. Within these masks, a pixel value of 1 signifies the presence of an object, while a value of 0 denotes the background. *F* represents the fitting of the ellipse of the mask, and *A* represents the area of the ellipse.

Contour points are extracted to refine the mask image. The fitEllipse method of OpenCV (version 4.8.0) is then used to obtain the ellipse center coordinates, major and minor axis lengths, and rotation angle. The fitEllipse method utilizes least squares to minimize the sum of distances from all contour points to the ellipse, thereby fitting the optimal ellipse. The CTR is then calculated based on the ratio of the areas of the two ellipses.

### Clinical validation

Three sonographers with varying levels of clinical experience—junior (1 year of prenatal screening), intermediate (5 years), and senior (10 years)—executed manual measurements on 100 randomly selected fetal four-chamber view ultrasound images from the test set, utilizing manual tracing techniques. The parameters measured were CAx and CTR. These ultrasound images were input to our trained AI model for automated computations with identical parameters. Manual sonographers and automated AI measurements were archived to enable comparative analysis.

### Statistical analysis

The mean Dice coefficient (mDice) and mean Intersection over Union (mIoU) are widely used and accepted in the field of medical image segmentation, and were employed to evaluate the accuracy of the fetal four-chamber view segmentation model.

We use mDice—which provides a measure of the spatial correspondence as well as the overlap between model-predicted and ground-truth segmentation—to compare region similarity in sample spaces. It is defined as twice the size of the overlapping area between the predicted and accurate segmentation divided by the total size of both segmented regions:

7 $$mDice=\left(\frac{1}{n}\right)\ast {\sum }_{i=1}^{\text{n}}\frac{2\times \left|{x}_{i}\cap {y}_{i}\right|}{\left|{x}_{i}\right|+\left|{y}_{i}\right|},$$

where $n$ is the number of classes, ${x}_{i}$represents the predicted segmentation for the $i$ class, and ${y}_{i}$represents corresponding ground-truth segmentation. The Dice coefficient ranges from 0 to 1, with a value closer to 1 indicating higher segmentation quality.

The mIoU is used to evaluate image segmentation quality. It is the average ratio of the intersection over the union between the predicted and ground-truth segmented regions, particularly useful for multiclass segmentation tasks because it calculates the average segmentation score across all classes, offering a balanced assessment of the model’s performance. It is calculated as

8 $$mIoU\,\; = \,\;\left( {{1 \over n}} \right)\, * \,\;\sum\limits_{i = 1}^n {\,\left( {{{\left| {{X_i} \cap \;{Y_i}} \right|} \over {\left| {{X_i} \cup \;{Y_i}} \right|}}} \right)} ,$$

where $n$ is the number of classes, ${X}_{i}$ is the segmentation for the $i$ class predicted by the model, and ${Y}_{i}$ is the corresponding ground-truth segmentation. The mIoU values range from 0 to 1, with larger values indicating higher overall segmentation accuracy.

These metrics are particularly suitable for evaluating the accuracy of our fetal four-chamber view segmentation model, as they consider both true- and false-positive predictions, and provide a comprehensive assessment of a model’s performance across multiple anatomical structures. Moreover, these widely adopted metrics enable direct comparison of our model with other methods.

The normality of the measured values from physicians with differing years of experience and the AI-based model was assessed using the Shapiro‒Wilk test. For data conforming to a normal distribution (*P* > 0.05), a paired sample t test was used to analyze the mean differences between physician and AI measurements. For non-normally distributed data (*P* ≤ 0.05), the Wilcoxon signed-rank test was employed to evaluate the differences.

To quantify the agreement between manual measurements obtained by expert sonographers and automated measurements obtained by the AI-based model, the intraclass correlation coefficient (ICC) and Bland‒Altman plots were used for statistical analysis. The ICC assesses reproducibility by determining the correlation between measurements. Bland–Altman plots graphically represent the agreement between two quantitative measurements by plotting the difference between the two measurements against their mean.

All statistical analyses were conducted using R language scripts in RStudio (version 4.3.2) and Python (version 3.9.0), with a significance level of α = 0.05.

## Results

### General results

A total of 1,442 fetal four-chamber views were obtained, 359 of which were excluded owing to inadequate image quality or incomplete fetal four-chamber views. The remaining 1,083 images revealed a mean gestational age of 25 ± 4 weeks (18–32 weeks). The remaining images were divided into training/validation and test sets at an 8:2 ratio. The training/validation set included 867 images for model development, and the test set comprised 216 images, which were used to assess model performance (Table 3). From this test set, 100 images were randomly selected for clinical validation by sonographers.

**Table 3:** Numbers of labels included in training and test sets

Label	Training/Validation	Test	Label	Training/Validation	Test
LA	867	216	LV	867	216
RA	867	216	RV	867	216
IVS	867	216	IAS	867	216
LVW	867	216	RVW	867	216
LL	867	216	RL	867	216
DAO	867	216	SP	867	216
RIB	1644	351	HA	867	216
			TA	867	216

*LV: left ventricle; LA: left atrium; RA: right atrium; RV: right ventricle; IVS: interventricular septum; IAS: interatrial septum; LVW: left ventricular wall; RVW: right ventricular wall; LL: left lung; RL: right lung; DAO: descending aorta; SP: spine; RIB: rib; HA: heart area; TA: thorax area

### Segmentation results

The nnU-NetV2, as developed in this study, attained an mDice value of 87.11, and mIoU was 77.68 (Table 4).

**Table 4:** Dice coefficient and intersection over union (IoU) of each label

Method	LV	LA	RA	RV	IVS	IAS	LVW	RVW	LL	RL	DAO	SP	RIB	HA	TA	mDice	mIoU
Unet [33]	83.06	83.27	87.06	76.49	79.08	53.19	73.94	69.69	80.78	82.34	78.89	83.96	67.53	31.15	26.03	70.43	-
	71.03	71.34	77.08	61.93	65.40	36.23	58.66	53.48	67.76	69.98	65.13	72.35	50.98	18.45	14.96	-	56.98
Unet++ [35]	84.02	84.02	87.48	79.31	80.22	54.82	75.34	70.96	81.96	83.58	79.47	84.69	68.20	34.68	27.30	71.74	-
	72.44	72.45	77.75	65.73	66.79	37.76	60.43	54.99	69.44	71.79	65.94	73.44	51.74	20.98	15.81	-	58.50
Deeplabv3+ [32]	89.75	90.52	93.54	87.33	82.41	60.00	82.74	81.51	93.35	92.41	83.55	91.79	**78.47**	46.71	59.29	80.89	-
	81.41	82.67	87.86	77.50	70.08	42.86	70.56	68.79	87.53	85.89	71.75	84.82	**64.57**	30.47	42.14	-	69.93
SAN [34]	**91.21**	91.78	93.30	**89.07**	**86.82**	64.90	**83.50**	**81.89**	**93.50**	92.86	87.68	92.44	78.34	45.22	62.47	82.33	-
	**83.83**	84.81	87.44	**80.30**	**76.71**	48.04	**71.67**	**69.33**	**87.79**	86.68	78.06	85.94	64.40	29.22	45.43	-	71.98
nnU-netV2	90.12	**92.42**	**94.11**	88.21	85.59	**72.92**	81.13	81.05	92.06	**93.23**	**89.91**	**93.91**	77.18	**88.19**	**86.67**	**87.11**	-
	82.01	**85.91**	**88.88**	78.91	74.80	**57.39**	68.25	68.14	85.29	**87.32**	**81.67**	**88.51**	62.84	**78.87**	**76.48**	-	**77.68**

*LV: left ventricle; LA: left atrium; RA: right atrium; RV: right ventricle; IVS: interventricular septum; IAS: interatrial septum; LVW: left ventricular wall; RVW: right ventricular wall; LL: left lung; RL: right lung; DAO: descending aorta; SP: spine; RIB: rib; HA: heart area; TA: thorax area

Bold values represents the maximum Dice or mIoU obtained for each structure in different models

### Visualization results

**Fig. 5:**
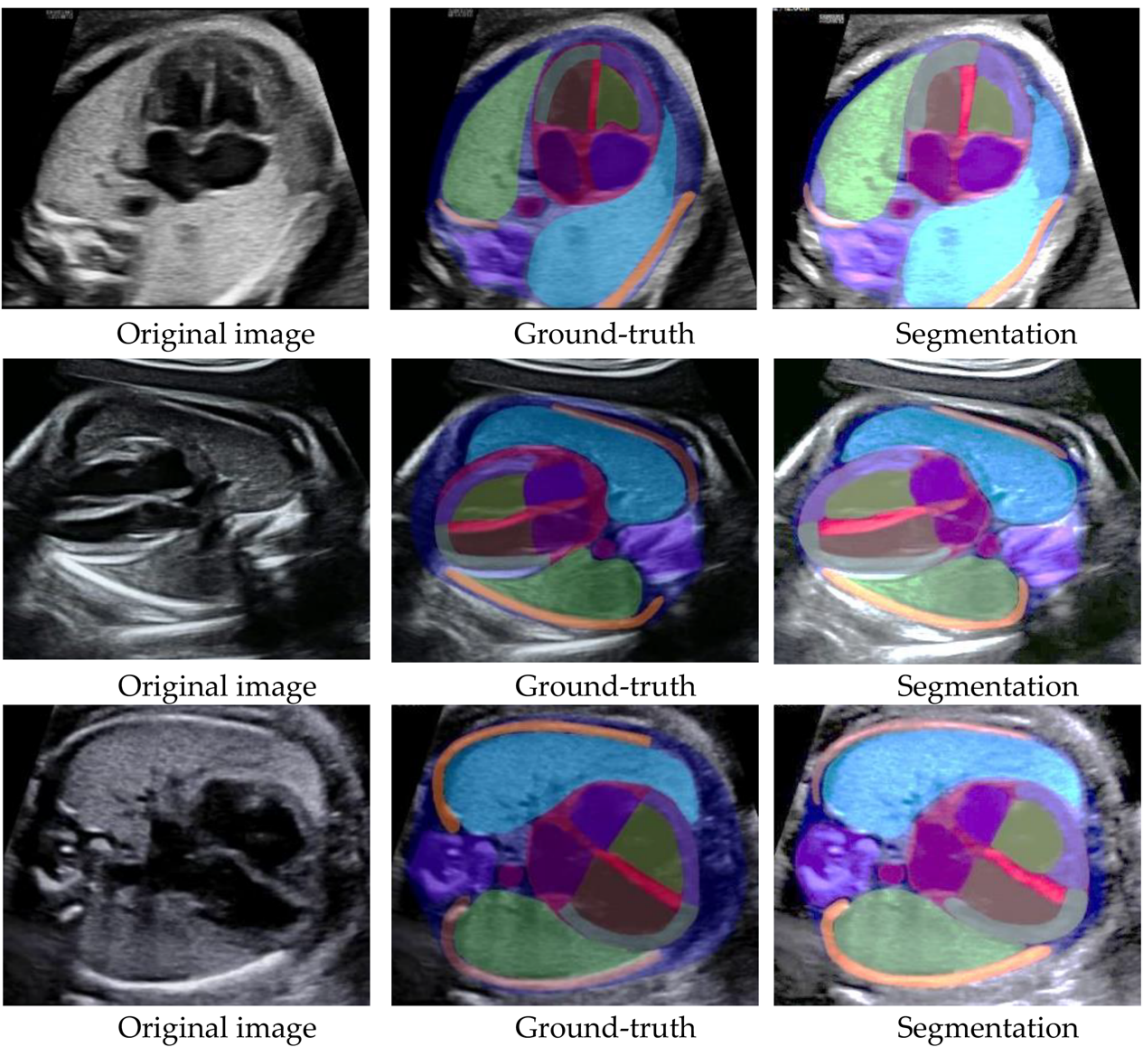
Visualization of segmentation results, showing original, manually annotated, and automatically segmented images for apical fetal four-chamber view, parasternal fetal four-chamber view, and basal fetal four-chamber view

**Fig. 6:**
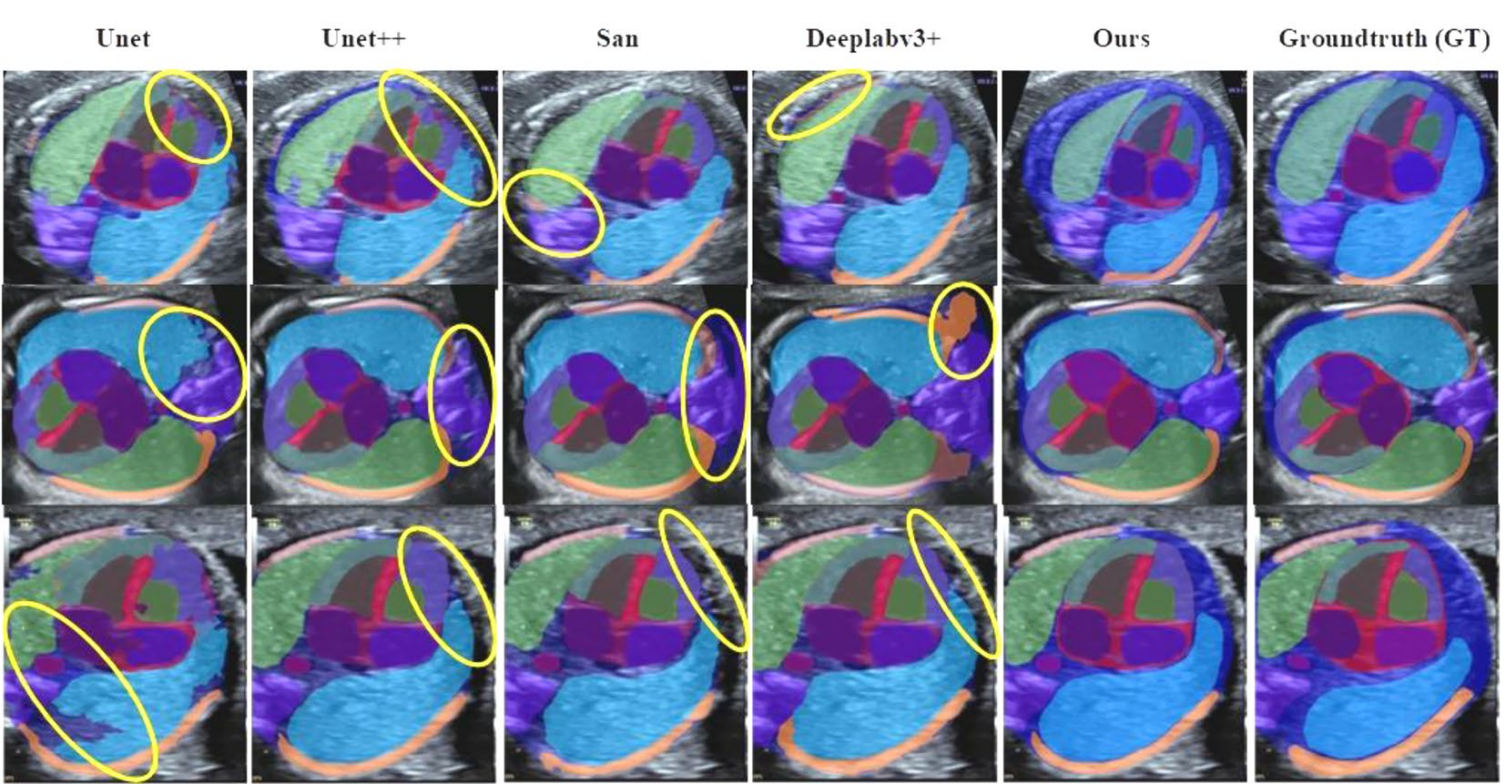
Visualization comparison. Yellow ellipses mark obvious differences between other models and ground-truth

The nnU-NetV2 effectively segmented all labels, with smooth contours and the absence of jagged edges (Fig. 5). Its visual segmentation is much closer to the ground-truth, as detailed in Fig. 6, where yellow ellipses highlight visible differences between the other models and the ground-truth.

### Expert vs. AI-based model measurement concordance analysis

**Fig. 7:**
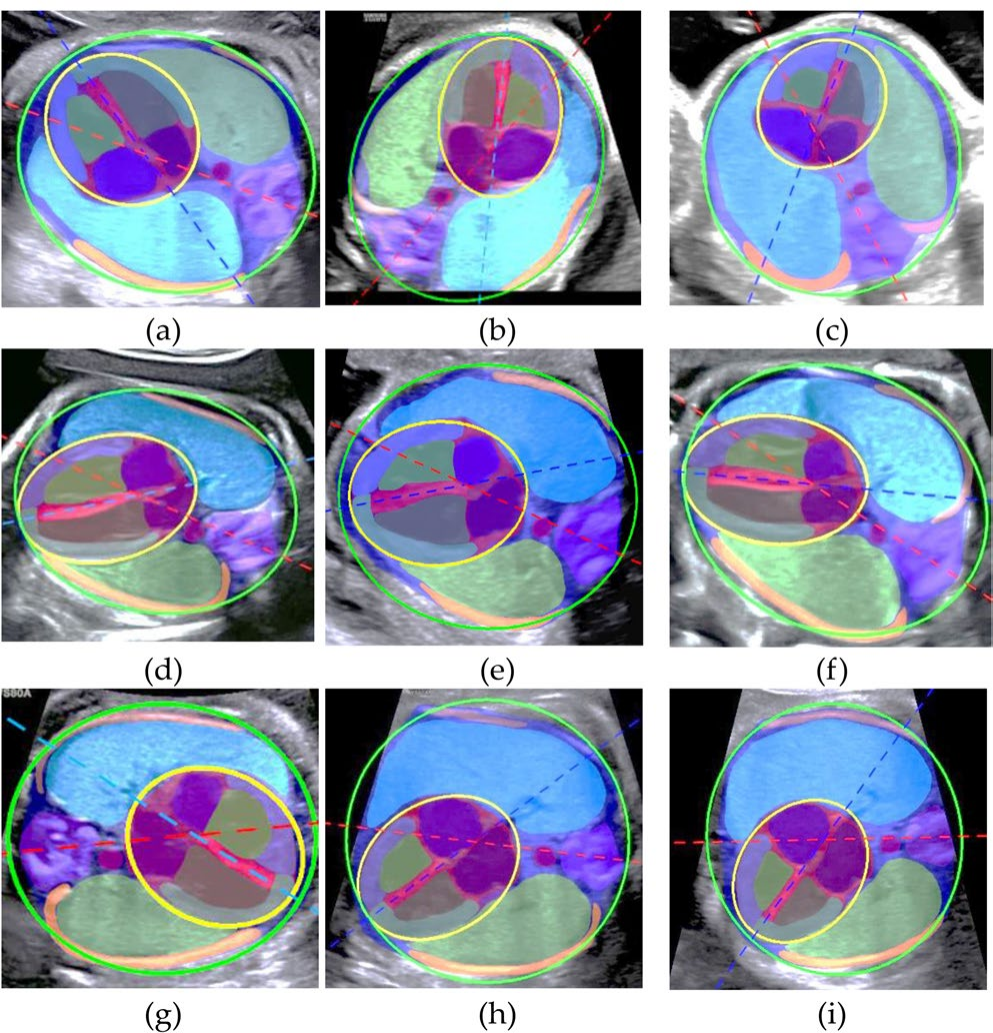
AI-based model measurements of cardiac parameters from various positions. (**a**)–(**c**) Apical fetal four-chamber view; (**d**)–(**f**) Parasternal fetal four-chamber view; (**g**)–(**i**) Basal fetal four-chamber view

Table 5 presents the CAx and CTR measurements obtained by the AI-based model and the three sonographers. Statistical analysis revealed significant differences in CAx measurements between the AI-based model and the sonographers (*P* < 0.05), while no significant differences were observed in CTR measurements (*P* > 0.05). AI-based model measurements of cardiac CAx and CTR visualization at different locations are shown in Fig. 7. The ICCs between the senior sonographers and the AI-based model were 0.83 for CAx, and 0.81 for CTR. The ICCs between intermediate sonographers and the AI-based model were 0.73 for CAx, and 0.81 for CTR. ICCs between junior sonographers and the AI-based model were 0.68 and 0.75 for CAx and CTR, respectively (Table 6).

**Table 5:** Cardiac axis and cardiothoracic ratios measured by sonographers with different levels of clinical experience and by AI

Operator	CAx			
	M ± SD	RANGE	Normality test	Vs. AI
Senior	33.19°±7.99°	7.9°∼ 54.6°	*P* = 0.2106	*P* = 0.01397
Intermediate	34.04°±9.34°	8.6°∼ 62.2°	*P* = 0.1401	*P* = 0.001526
Junior	35.86°±8.76°	5.7°∼ 57.1°	*P* = 0.01056	P*= 3.364e-08
AI	32.03°±8.19°	10.2°∼ 50.9°	*P* = 0.2726	
	**CTR**			
Senior	0.31 ± 0.05	0.21 ∼ 0.43	*P* = 0.05688	P*= 0.8565
Intermediate	0.31 ± 0.04	0.22 ∼ 0.42	*P* = 0.05167	P*= 0.148
Junior	0.31 ± 0.05	0.19 ∼ 0.44	*P* = 0.4415	P*= 0.09368
AI	0.31 ± 0.04	0.23 ∼ 0.45	*P* = 0.01863	

*: Wilcoxon signed-rank test

**Table 6:** Intra-observer variability (ICC) between sonographers of varying experience levels and AI.

Operator	CAX ICC (95%CI)	CTR ICC (95%CI)
Senior	0.83(0.75 ∼ 0.88)	0.81(0.74 ∼ 0.87)
Intermediate	0.73(0.61 ∼ 0.82)	0.81(0.73 ∼ 0.87)
Junior	0.68(0.39 ∼ 0.82)	0.75(0.66 ∼ 0.83)

**Fig. 8:**
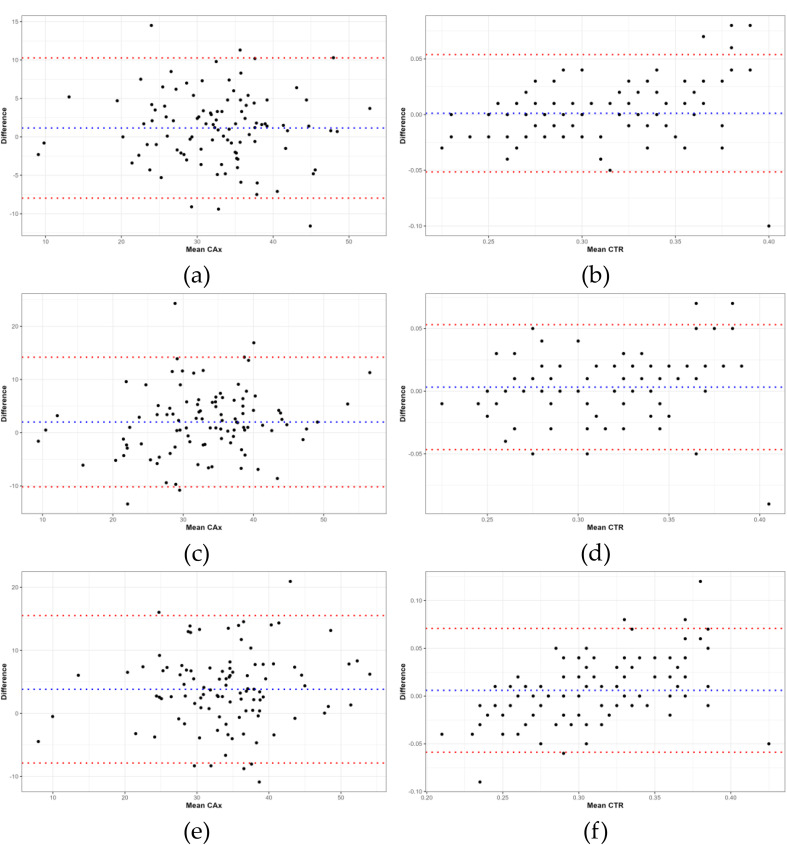
Bland–Altman plots exhibiting intra-observer variability for cardiac axis (CAx) and cardiothoracic ratio (CTR) measurements. Blue dotted line: mean difference; red dotted line: 95% limits of agreement. (**a**) CAx measurements by senior sonographer; (**b**) CTR measurements by senior sonographer; (**c**) CAx measurements by intermediate sonographer; (**d**) CTR measurements by intermediate sonographer; (**e**) CAx measurements by junior sonographer; (**f**) CTR measurements by junior sonographer

Bland–Altman analysis was utilized to evaluate the concordance between the AI-based model and sonographers with varying levels of clinical experience in CAx measurements. The senior sonographer exhibited a mean bias of 1.15° with 95% confidence intervals (CIs) of 0.25–2.06° and 95% limits of agreement (LoA) from − 7.97 to 10.28°. The intermediate sonographer had a mean bias of 2.01° (95% CI: 0.80 to 3.21°), with a 95% LoA between − 10.18 and 14.19°. The junior sonographer’s mean bias was 3.83° (95% CI: 2.67°-4.98°), with a 95% LoA ranging from − 7.87 to 15.52°. For CTR measurements, the Bland–Altman plots indicated the following levels of agreement between the AI-based model and sonographers with various levels of clinical experience. The senior sonographer exhibited a mean bias of 0.0012 (95% CI: −0.0040 to 0.0064) and a 95% LoA ranging from − 0.0515 to 0.0538. The intermediate sonographer had a mean bias of 0.0032 (95% CI: −0.0017 to 0.0082), with a 95% LoA between − 0.0466 and 0.0530. The junior sonographer’s mean bias was 0.0060 (95% CI: −0.0004 to 0.0124), with a 95% LoA ranging from − 0.0588 to 0.0708 (Fig. 8).

## Discussion

In recent years, the use of artificial intelligence to automate prenatal ultrasound measurements has been an active research area [8, 28]. As early as 2008, deep learning was used to automatically measure multiple fetal anatomical parameters, including the biparietal diameter, head circumference, and long bone length, achieving comparable measurements to those of skilled sonographers, and reducing the workload by approximately 75% [36]. Most related studies still focus on automating these conventional parameters, with remarkable progress [2, 29, 30]. However, research on quantifying fetal cardiac parameters has been relatively limited [1, 26].

The four-chamber view is the most critical plane in fetal echocardiography screening; in this view, authoritative guidelines emphasize evaluating the CAx and cardiothoracic area ratio [37]. Moreover, CAx and CTR measurements depend heavily on the sonographer’s expertise and experience. In busy hospitals, assessments are often performed by sonographers of varying skill levels [38]. Significant measurement errors increase sonographer workloads, waste resources, prompt unnecessary examinations, escalate maternal anxiety, and can lead to missed diagnoses [2].Research on automating cardiac parameter quantification is indispensable and clinically valuable, which is the motivation for this study.

The accurate segmentation of the fetal four-chamber view achieved by our AI-based method lays the foundation for further analysis of images and the development of advanced diagnostic tools. By enabling the automated measurement of critical cardiac parameters, such as the CAx and cardiothoracic ratio, our approach provides valuable insights into fetal cardiac health, and facilitates the detection of potential abnormalities. Moreover, the segmentation masks generated by our method can serve as starting points for the extraction of additional cardiac features and the development of comprehensive diagnostic models. The integration of these advanced features with machine learning algorithms holds promise for the early detection and risk stratification of congenital heart defects. Furthermore, our method’s segmentation capabilities open possibilities for the creation of intelligent tools that can assist clinicians in decision-making, treatment planning, and patient communication. The potential for integration with other imaging modalities further enhances the appropriateness of our method for a holistic assessment of fetal cardiac health.

The optimal gestational age for fetal echocardiography is 18–22 weeks [4]. However, evaluation of the four-chamber view may be needed for up to 30 weeks’ gestation in clinical practice. Therefore, the gestational ages of fetuses used to develop our model spanned from 18 to 32 weeks. Notably, the rib training/validation set had 1,644 images, and the test set had 351 images because the collected views varied, showing one, two, or incomplete ribs. All images were counted and annotated despite having the largest training dataset in the label we trained the model on; Dice and IoU for ribs were still suboptimal due to variability in rib presentation.

This study demonstrates an AI-based model that uses the nnUnet-V2 architecture for fetal four-chamber section segmentation. The results show that the AI-based model accurately identifies and segments 15 key anatomical landmarks, and that its performance is closer to that of a sonographer’s manual annotation with nnUnet-V2 than with four state-of-the-art semantic segmentation models. In addition, the nnUnet-V2-based model automatically calculates CAx and CRT in fetal four-chamber views, highlighting the potential of deep learning in clinical practice. Whether in apical, parasternal, or basal views, the model effectively segments and measures the results.

We quantitatively compared nnU-NetV2 with four state-of-the-art semantic segmentation methods, including the latest SAN methods, which were consistently outperformed by nnU-netV2 in most evaluated classes. Notably, for LV, SAN had a Dice score and an IoU of 91.21 and 83.83%, respectively, but nnU-netV2 achieved a slightly lower yet competitive Dice score and an IoU of 90.12 and 82.01%, respectively. Impressively, in the RL category, nnU-netV2 had a Dice score and an IoU of 93.23 and 87.32%, respectively, exceeding SAN at 92.86 and 86.68%, respectively. Across all classes, nnU-netV2 showed a significant improvement, with an mDice score and mIoU of 87.11 and of 77.68%, respectively, compared to that of SAN’s 82.33 and 71.98%, respectively. The analysis explores each class in detail, particularly emphasizing nnU-netV2’s superior performance in segmenting complex anatomical structures. Notable improvements were observed for IAS and LA, with Dice score enhancements of 8.02% and 0.64%, respectively, compared to the SAN. These results suggest that nnU-netV2 is particularly effective at segmenting intricate anatomical features.

In light of the above, sonographers must manually segment structures such as the spine, septum, ribs, and thorax when measuring CAx and CRT prenatally [3, 4]. Identifying these boundaries can be challenging for novices. Factors such as fetal position, amniotic fluid volume, and movement further complicate measurements [39]. Moreover, nnU-netV2 can measure the CAx and CRT in fetal four-chamber views at different positions, as shown in Fig. 7. Computing CRT requires separate heart and thorax delineation, often requiring 2–3 min to obtain satisfactory results. Computation can be much faster using the nnU-NetV2 model, and the clinical application of this approach could reduce the workload of sonographers, give doctors more time with patients, and potentially mitigate doctor-patient conflicts.

There were no statistically significant differences in the CTR (*P* > 0.05) between the three sonographers with different levels of clinical experience and the AI-based model. This indicates that the overall measurement accuracy of the AI-based model was comparable to that of physicians. ICC analysis revealed consistency levels. The senior (ICC = 0.81) as well as the intermediate (ICC = 0.81) sonographers demonstrated good consistency with the AI-based model. The junior radiologist had slightly lower consistency (ICC = 0.75), but was still within the acceptable range. Despite different clinical experiences, the sonographers’ CTR measurements were consistent with those of the AI-based model. Bland–Altman analysis further validated the minor differences in the CTR between the AI model and sonographers. The senior sonographer had a slight mean deviation (0.0012), and the 95% CI (− 0.0040 to 0.0064) and LoA (− 0.0515 to 0.0538) indicated that most deviations were within a tiny range. The intermediate sonographer exhibited a similar pattern, with a mean deviation of 0.0032 and good consistency. The junior sonographer had a slightly larger mean deviation (0.0060). Although the 95% CI was zero, indicating no statistically significant difference from the AI-based model, the range of disagreements was slightly broader than that of the senior and intermediate sonographers, showing a slightly lower consistency.

When analyzing CAx measurements, the AI-based model showed statistically significant differences compared to sonographers with varying degrees of clinical experience (*P* < 0.05). The measurement consistency was highest between the senior sonographer and AI (ICC = 0.83), followed by the intermediate sonographer (ICC = 0.73) and the junior sonographer (ICC = 0.68). This reflects greater consistency between AI and more experienced sonographers for CAx. Bland–Altman analysis also showed that the AI-based model had the most minor mean deviation from the senior sonographer (1.15°), indicating the best consistency. Furthermore, the intermediate sonographer had a 2.01° mean deviation, showing intermediate consistency. The junior sonographer had the most significant deviation (3.83°), indicating the worst consistency. Despite systematic bias, the overall consistency of AI with more experienced sonographers was greater for CAx measurements.

A noteworthy innovation of the present study was the development of an AI-based model using the nnU-NetV2 architecture to enable automated segmentation and measurement of fetal four-chamber views in mid-to-late gestation. This approach facilitated accurate quantification of CAx and CTR, which had not been previously automated. The model showed robust agreement with manual measurements by experienced sonographers. The application of this technology could improve clinical workflow efficiency while maintaining diagnostic accuracy. However, limitations exist regarding model validation with constrained sample sizes and the need for multicenter assessments. Although the current training dataset supported preliminary model development, future studies leveraging larger multicenter sample sizes are imperative to validate the generalizability and expansive clinical utility of the model. This will be an important step in advancing automated echocardiographic analysis, providing more precise and standardized screening and diagnostic tools for fetal cardiac abnormalities.

## Conclusion

In this study, we developed an AI-based model using the nnU-NetV2 architecture for automatic segmentation of the fetal four-chamber view and measurement of CAx and CTR. The model successfully identified and segmented 15 critical anatomical labels in fetal four-chamber views, enabling the automated computation of CAx and CTR. The model’s performance was excellent, with mDice and mIoU of 87.11 and 77.68%, respectively, which indicated accurate recognition of anatomical structures. The measurements obtained by the AI-based model demonstrated strong agreement with those of sonographers, thereby highlighting its potential diagnostic value.

Our findings suggested that the AI-based model could provide meaningful diagnostic support to sonographers with varying levels of expertise. In addition, the model could serve as a robust training and mentoring tool for less experienced sonographers, helping them to improve their fetal echocardiography skills. The model could help reduce the workload of experienced sonographers and increase productivity by providing accurate and consistent measurements. As such, integrating this technology into clinical practice could enhance the standardization of prenatal cardiac screening and facilitate earlier detection and treatment of abnormalities.

The AI-based model developed in this study could have numerous applications. By leveraging the segmentation model, additional cardiac parameters could be measured to comprehensively evaluate fetal cardiac health. Furthermore, the highly scalable nature of the model enables the development of customized models for different cardiac planes and the identification and analysis of plane-specific structures, ultimately improving diagnostic capabilities.

Despite the promising results, this study had certain limitations. First, the dataset used for training may not fully represent the entire spectrum of anatomical variations and pathologies encountered in clinical practice. Expanding the dataset to include a more diverse range of cases could enhance the model’s robustness and generalizability. Second, the model’s decision-making process may not be easily interpretable by clinicians, which could hinder its adoption in clinical settings. Incorporating techniques for explainable AI could help improve the transparency and trustworthiness of the model. Third, because the current implementation focused on offline analysis, adapting the model for real-time performance during live ultrasound examinations would require further optimization and integration with ultrasound systems. Finally, our model was specifically designed for the analysis of the fetal four-chamber view and the measurement of CAx and CTR; therefore, extending the model’s capabilities to other cardiac views and additional measurements would provide a more comprehensive evaluation of fetal cardiac health.

In the future, our goal is to harness the power of artificial intelligence to streamline and standardize the screening and diagnosis of congenital heart defects. By improving the accuracy of early detection, we aim to enhance patient outcomes through timely intervention. The integration of AI-driven models into routine prenatal care could revolutionize fetal echocardiography, making it more intelligent and standardized across various healthcare settings. This could ensure consistent, high-quality fetal cardiac care, regardless of geographic location or practitioner expertise.

Our study demonstrated the successful development of an AI-based model for automatic segmentation and measurement of fetal four-chamber views. The model achieved excellent performance, with mDice and mIoU of 87.11 and 77.68%, respectively, in addition to showing strong agreement with sonographer measurements. These findings highlighted the model’s potential to provide meaningful diagnostic support across different levels of expertise, standardize prenatal cardiac screening, and improve early detection of abnormalities. Despite limitations, the integration of this technology into clinical practice could ultimately enhance patient outcomes. Future research should address these limitations, further validate the model, explore additional applications, and develop customized models for different cardiac planes to maximize its diagnostic capabilities and clinical impact.

## Data Availability

Data is provided within the manuscript . The detail of data has been described within the manuscript.
